# Factors associated with left ventricular mass during disease modifying antirheumatic drug therapy in patients with rheumatoid arthritis: the Joint Heart study

**DOI:** 10.1016/j.ijcrp.2025.200521

**Published:** 2025-10-01

**Authors:** Anja Linde, Eva Gerdts, Bjørg T. Fevang, Arve Ulvik, Per M. Ueland, Klaus Meyer, Ester Kringeland, Helga Midtbø

**Affiliations:** a Centre for Research on Cardiac Disease in Women, Department of Clinical Science, University of Bergen, Bergen, Norway; b Norwegian Research Centre for Women's Health, Oslo University Hospital, Oslo, Norway; c Department of Heart Disease, Haukeland University Hospital, Bergen, Norway; d Department of Rheumatology, Haukeland University Hospital, Bergen, Norway; e Bevital AS, Bergen, Norway; f Department of Clinical Science, University of Bergen, Bergen, Norway

**Keywords:** Rheumatoid arthritis, Inflammatory markers, DMARDs, Echocardiography, Left ventricular mass index

## Abstract

**Background:**

We explored factors associated with left ventricular (LV) mass index during biological (b) or targeted synthetic (ts) disease modifying antirheumatic drug (DMARD) therapy in patients with rheumatoid arthritis (RA).

**Methods:**

Eighty-three outpatients with RA (age 55 ± 12 years, 71% women) with an indication for b/ts DMARD therapy were examined with echocardiography at baseline and after a mean follow-up of 22 months. LV mass was calculated according to guidelines and indexed for height^2.7^.

**Results:**

At baseline, 37% had hypertension, 6% diabetes, 21% obesity, and 100% were using b/ts DMARDs. During follow-up, 17% discontinued b/tsDMARD treatment. The LV mass index remained unchanged during follow-up (33.1 ± 8.1 g/m^2.7^ vs. 33.5 ± 7.3 g/m^2.7^, p = 0.57, mean change 0.3 ± 4.9 g/m^2.7^). Lower LV mass index at follow-up was observed in patients using bDMARDs at follow-up (31.7 ± 6.2 g/m^2.7^ vs. 36.6 ± 8.9 g/m^2.7^, p = 0.001). In multivariable linear regression analyses, use of bDMARDs (β −0.22, p = 0.03) at follow-up were associated with lower LV mass index at follow-up, independent of C-reactive protein (CRP), age, sex, and obesity at baseline. Obesity at baseline (β 0.39, p < 0.001) was associated with a higher LV mass index both at baseline and follow-up. Higher CRP at baseline was associated with higher LV mass index at baseline (β 0.31, p = 0.001), but not at follow-up.

**Conclusion:**

In patients with RA on DMARD treatment, the mean LV mass index remained stable during 22 months of follow-up. Obesity was the strongest factor associated with higher LV mass index, while use of bDMARD throughout the study period was associated with lower LV mass index.

## Highlights

• We investigated factors associated with left ventricular (LV) mass in rheumatoid arthritis (RA).
• Mean LV mass did not change during two years of follow-up.
• Patients with obesity had greater LV mass.
• Continuous use of immunosuppressive treatment was associated with lower LV mass.

## Graphical abstract

**Figure:**
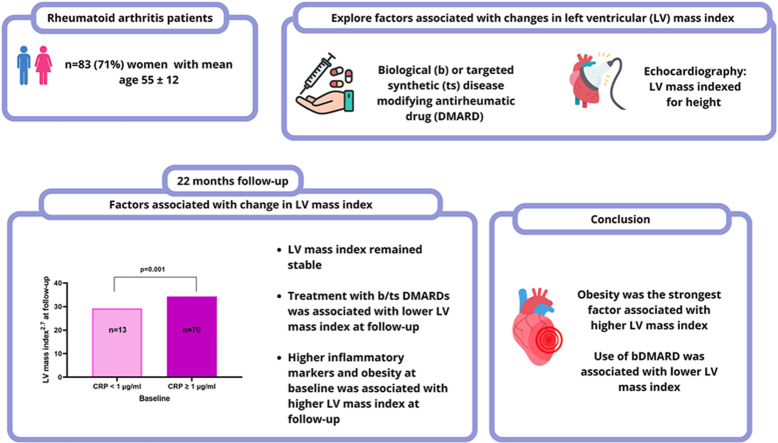

## Abbreviations

b: Biological
BMI: Body mass index
BP: Blood pressure
CMR: Cardiac magnetic resonance imaging
CRP: C-reactive protein
CVD: Cardiovascular disease
DAS28: Disease Activity Score in 28 joints
DMARD: Disease modifying antirheumatic drug
IQR: Interquartile range
LV: Left ventricular
RA: Rheumatoid arthritis
SAA: Serum amyloid A
ts: Targeted synthetic
TNF-α: Tumour necrosis factor alpha

## 1 Introduction

Rheumatoid arthritis (RA) is an autoimmune disease characterized by systemic inflammation and increased risk of cardiovascular disease (CVD). The role of chronic inflammation for the development of atherosclerotic CVD is well documented [1,2]. However, chronic inflammation does not only promote atherosclerosis but also alterations in left ventricular (LV) structure through induction of myocardial hypertrophy and fibrosis [3,4]. Abnormal LV structure, such as increased LV mass and LV hypertrophy, are strong predictors of future cardiovascular events and associated with volume or pressure over-load conditions [5,6]. However, even normotensive RA patients have been demonstrated to have an increased prevalence of abnormal LV structure [7]. European guidelines recommend searching for organ damage, such as increased LV mass, to guide initiation of early preventive measures like antihypertensive treatment [8]. Still, previous cross-sectional studies have yielded inconsistent results regarding whether LV mass is increased in patients with RA compared to controls and there is a lack of longitudinal echocardiographic studies [9,10].

Treatment with disease modifying antirheumatic drugs (DMARDs) reduces both systemic and joint inflammation in RA [11]. However, few longitudinal studies have reported change in LV mass during treatment with biological (b) or targeted synthetic (ts) DMARD therapy [[12], [13], [14]]. Using cardiac magnetic resonance imaging (CMR), Plein et al. found an increase in LV mass in 81 patients with RA over 2 years treatment with etanercept [13]. In contrast, Kobayashi et al. reported a reduction in LV mass during 1 year of treatment with tocilizumab [12]. In clinical practice echocardiography is the preferred method for assessment of LV mass because of cost and availability, however the effect of b/tsDMARD treatment on change in LV mass has been less studied in patients with RA using echocardiography. In the JointHeart study, patients with RA underwent echocardiographic assessment at the initiation of b/tsDMARD treatment and again after an average follow-up of 22-months. The study aimed to determine if treatment with b/tsDMARDs was associated with lower LV mass index and to identify factors associated with LV mass index during therapy in patients with RA.

## 2 Methods

### 2.1 Study population

This was a longitudinal observational study. Patients with RA who were accepted for initiation or change in b/tsDMARD therapy from 2018 to 2020 at the Department of Rheumatology, Haukeland University Hospital, Bergen, Norway were consecutively invited to participate in the study [15]. Cardiac disease, defined as previous myocardial infarction, previous cardiac surgery or heart failure, current severe psychiatric illness, and expected low compliance were criteria of exclusion. Patients underwent baseline cardiovascular assessments from 2018 to 2020 and follow-up assessments from 2019 to 2021 with some delay because of the covid-19 pandemic, mean follow-up length was 22 months. A total of 113 patients with RA were invited to participate in the study of which 104 accepted the invitation. Of these, 88 returned for the follow-up visit. Five patients were excluded in the current analysis due to lack of biobank samples, leaving 83 patients for the present analyses. There was no statistically significant difference in the age, gender, CRP levels or prevalence of obesity between the participants that returned for the follow-up visit and those who did not. The study was approved by the Research Ethics Committees of Western Norway (REK 2017–02257) and conducted in accordance with the Declaration of Helsinki. All the study participants signed a written informed consent.

### 2.2 Echocardiography

Echocardiography was done using a Vivid E9 (GE Healthcare, Horten, Norway) scanner following a standardized protocol. Off-line analyses of digital echocardiographic images were performed at workstations equipped with the Image Arena (TomTec, Unterschleissheim, Germany) software, at the Bergen Echocardiographic Core Laboratory, University of Bergen. The joint guidelines from the European Association of Cardiovascular Imaging and American Society of Echocardiography were followed when performing quantitative echocardiographic analyses [16]. All images were first analysed by a junior reader (AL) and thereafter proofread by a senior reader (HM) as recommended for echocardiographic studies [17]. Reproducibility of echocardiographic measurements from our laboratory has been documented previously [18,19].

LV wall thicknesses and dimensions were measured in the two-dimensional parasternal long-axis view. LV mass was calculated using a necropsy validated formula [20]. To avoid inaccuracies in indexation of LV mass in patients with overweight and obesity, LV mass was indexed for height in the allometric power of 2.7 [21,22]. LV hypertrophy was defined as LV mass index >50.0 g/m^2.7^ in men and >47.0 g/m^2.7^ in women, in accordance with guidelines [23]. Relative wall thickness was calculated by 2 x posterior wall thickness/LV internal diameter at end-diastole. If the relative wall thickness was ≥0.43, concentric LV geometry was considered present [23].

### 2.3 Analyses of inflammatory biomarkers

Blood samples were collected at the Biobank at the Department of Rheumatology, Haukeland University hospital, and stored at - 80 °C before further analyses at the Bevital laboratory, Bergen, Norway (www.bevital.no). The acute phase reactants C-reactive protein (CRP), serum amyloid A (SAA) as well as the marker of inflammation, calprotectin was measured using Matrix-Assisted Laser Desorption/Ionization Time-Of-Flight (MALDI-TOF) mass spectrometry [24].

### 2.4 Clinical assessment

*Cardiovascular risk factor assessment.* Self-reported medical history, including current medication was collected using a standardized questionnaire and quality assured against the hospital medical records by the study doctor. Height, body weight, and waist circumference were measured by a trained study nurse. Body mass index (BMI) was calculated as the weight in kilograms divided by the square of the height in meters (kg/m^2^). Obesity was defined as BMI ≥30 kg/m^2^ according to the criteria of the World Health Organization. Diabetes Mellitus was considered present if the patient used antidiabetic medication or had known diabetes. Smoking was classified as smoking or non-smoking.

*Blood pressure measurement.* Office blood pressure (BP) was measured three times with 1-min intervals after an initial 5-min of rest in the seated position using a regularly calibrated Welch Allyn Connex ProBP 3400 Digital BP device (Hill-Rom Holdings, Chicago, USA) or an Omron HEM-907 BP device (Omron Corporation, Kyoto, Japan). The average of the last two measurements was taken as the office BP [23]. Hypertension was defined as presence of systolic BP ≥140 mmHg or diastolic BP ≥90 mmHg or use of antihypertensive drugs in the individual patient.

*RA Disease activity.* The Disease Activity Score in 28 joints using C-reactive protein (DAS28) was assessed at the Department of Rheumatology. Disease activity was classified based on DAS28 as follows; remission (DAS28 <2.6), low disease activity (DAS28 2.6 to 3.2), moderate disease activity (DAS28 3.3 to 5.1) and high disease activity (DAS28 >5.1) [25,26]. Information regarding the disease activity was extracted from the Norwegian Arthritis Registry (NorArthritis) [27]. Discontinuation of DMARD treatment was decided by the treating rheumatologist.

### 2.5 Statistical analysis

Statistical analyses and data management was performed using IBM SPSS version 29 (IBM, Almonk, NY, USA). Continuous data are reported as the means (standard deviation [SD]) for normally distributed variables, and categorical variables are reported as numbers (percentages). Non-normally distributed variables were reported as medians (interquartile range) and log transformed before inclusion in uni-and multivariable analyses. The paired-sample *t*-test, Wilcoxon signed-rank test or McNemar's test were used when comparing variables at baseline and follow-up as appropriate. Comparison between groups were done by the Student t-test. Uni-and multivariable associations were assessed by linear regression. Covariables with a p-value <0.01 in univariable analyses were tested in multivariable analyses. Multivariable linear regression models were done using an enter method and collinearity tools, and the results reported as multiple R^2^ for models and standardized beta coefficients (β) for individual variables. β is calculated as B/SDx, where β represent the change in the outcome variable associated with a one-standard deviation change in the independent variable (x). Normality of the regression models were assessed by probability plot of the residuals. A two-tailed p-value of <0.05 was considered statistically significant in all analyses.

## 3 Results

### 3.1 Clinical characteristics

At baseline, patients were on average 55 years old, 71% were women and the duration of RA was 10.6 years*.* The prevalence of hypertension was 37%, 21% were obese, and 6% had diabetes (Table 1). At baseline, 74% of patients had either moderate or high disease activity, and all used a b/tsDMARD.

**Table 1:** Clinical characteristics in the study population at baseline and follow-up.

	Baseline N = 83	Follow-up N = 83	P
**Clinical characteristics**			
Age, years	55 ± 12	57 ± 11	–
Women, n (%)	59 (71)	59 (71)	–
Body mass index, kg/m^2^	26.3 ± 5.3	26.9 ± 5.0	0.05
Obesity, n (%)	17 (21)	19 (23)	0.63
Waist circumference, cm	87.3 ± 14.3	92.9 ± 13.0	<0.001
Serum creatinine, μmol/L (median, IQR)	65 (57, 75)	68 (61, 75)	0.007
**RA characteristics**			
Disease duration, years	10.6 ± 8.1	12.4 ± 8.0	–
DAS28 score	3.9 ± 1.3	2.8 ± 1.2	<0.001
Number of swollen joints	4.7 ± 4.4	1.5 ± 2.7	<0.001
Number of tender joints	5.7 ± 6.7	3.0 ± 4.8	<0.001
bDMARDs, n (%)	61 (74)	53 (64)	0.10
tsDMARDs, n (%)	22 (27)	16 (19)	0.18
b/tsDMARDs, n (%)	83 (100)	69 (83)	<0.001
Methotrexate, n (%)	51 (61)	53 (64)	0.79
Prednisolone, n (%)	34 (41)	27 (33)	0.12
Current smoking, n (%)	14 (17)	10 (13)	0.38
Diabetes, n (%)	5 (6)	4 (5)	1.00
Use of statins, n (%)	2 (3)	3 (4)	1.00
Systolic BP, mmHg	129 ± 17	126 ± 18	0.005
Diastolic BP, mmHg	83 ± 10	78 ± 9	<0.001
Hypertension, n (%)	31 (37)	35 (42)	0.39
Use of antihypertensive drugs, n (%)	16 (20)	18 (22)	0.50
**Inflammatory biomarkers**			
CRP, median (IQR), μg/ml	4.6 (1.5, 14.4)	3.0 (1.1, 6.8)	0.001
Serum amyloid A, median (IQR), μg/ml	5.9 (2.4, 13.8)	3.3 (1.6, 7.9)	<0.001
Calprotectin, median (IQR), μg/ml	1.3 (0.9, 2.7)	0.8 (0.5, 1.6)	<0.001

BP: blood pressure; b/ts: biological/targeted synthetic; DMARDs: disease modifying antirheumatic drugs; CRP: C-reactive protein; DAS28: disease activity score in 28 joints; IQR: Interquartile.

range; RA: rheumatoid arthritis.

**Fig. 1:**
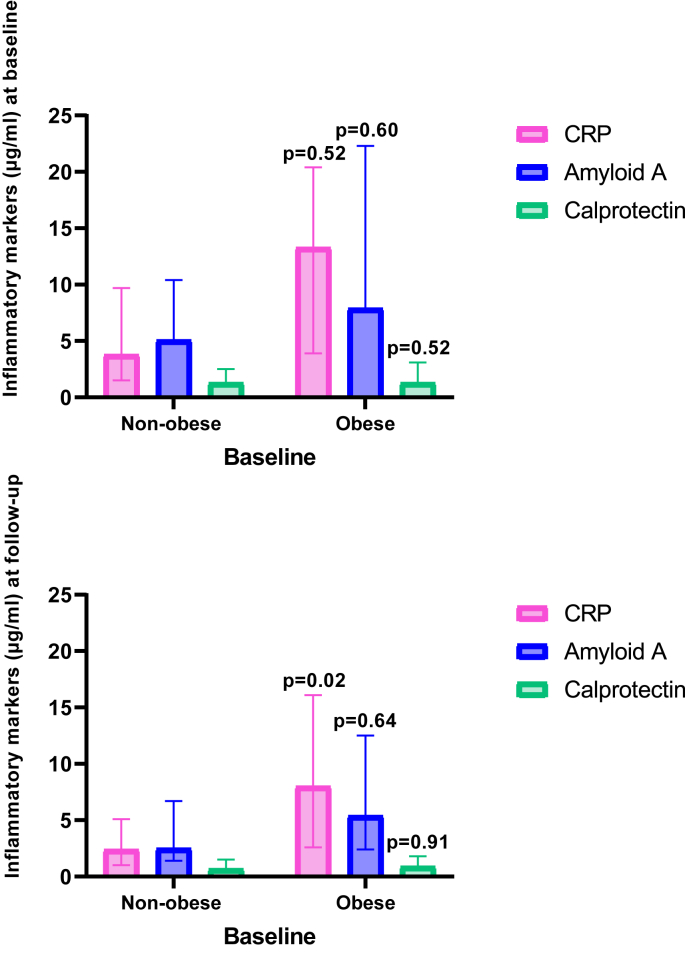
Inflammatory markers in patients with obesity and non-obese patients at baseline (panel A) and at follow-up (panel B).Panel A Panel B CRP: C-reactive protein. The bars represent the median, and the whiskers the interquartile range. The p-values refer to the difference between baseline and follow up in the individual marker.

During follow-up, the average BP decreased, while the prevalence of hypertension, obesity, smoking, and diabetes remained stable (Table 1). The proportion of patients using b/tsDMARDs was reduced to 83 % (Table 1). Although the disease activity was significantly lower at follow-up, 28 % of patients remained with moderate or high disease activity. The inflammatory biomarkers, SAA, CRP, and calprotectin, decreased from baseline to follow-up (Table 1). There was no difference in level of inflammatory markers at follow-up in patients using b/tsDMARDs compared to those that did not; however, DAS28 at follow-up was higher (3.7 ± 1.2 vs 2.7 ± 1.1, p = 0.001) in patients not using b/tsDMARDs at follow-up. Patients with obesity at baseline had higher CRP at follow-up compared to patients that were non-obese (Fig. 1).

### 3.2 Left ventricular mass index

**Fig. 2:**
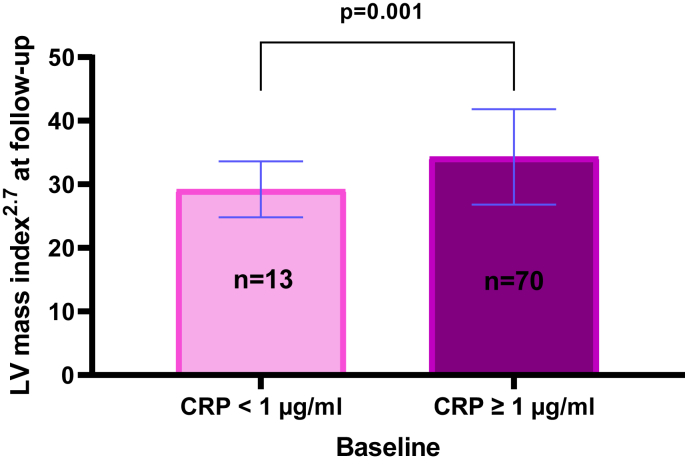
Association of baseline CRP level and LV mass index at follow-up. CRP: C-reactive protein. Results are presented as median, and the whiskers the interquartile range. The p-values refer to the difference in LV mass index between groups of patients with CRP <1 μg/ml and the group with CRP ≥1 μg/ml.

The average LV mass index did not change during the follow-up period, and the prevalence of LV hypertrophy was low both at baseline and follow-up (Table 2). Higher LV mass index at follow-up was observed in patients with high versus low disease activity at baseline (36.2 g/m^2.7^ vs. 29.6 g/m^2.7^, p = 0.046) and in patients having CRP level ≥1 μg/ml at baseline (34.3 g/m^2.7^ vs. 29.2 g/m^2.7^, p = 0.002) (Fig. 2). Lower LV mass index at follow-up was observed in patients using bDMARDs at follow-up (36.6 ± 8.9 g/m^2.7^ vs. 31.7 ± 6.2 g/m^2.7^, p = 0.001).

**Table 2:** Echocardiographic findings in the study population at baseline and follow-up.

	Baseline n = 83	Follow-up n = 83	P
Interventricular septum thickness at end diastole, cm	0.9 ± 0.15	0.9 ± 0.14	0.91
LV diameter at end diastole, cm	4.8 ± 0.48	4.8 ± 0.43	0.19
LV posterior wall thickness at end diastole, cm	0.8 ± 0.13	0.8 ± 0.11	0.53
LV mass index, g/m^2.7^	33.1 ± 8.1	33.5 ± 7.3	0.57
LV hypertrophy, n (%)	5 (6)	2 (2)	0.25
LV relative wall thickness, ratio	0.34 ± 0.06	0.33 ± 0.06	0.34
Concentric geometry, n (%)	9 (11)	5 (6)	0.34
Ejection fraction, %	63 ± 5	64 ± 5	0.26
Abnormal geometry, n (%)	12 (15)	7 (8)	0.23

LV: left ventricle.

Higher CRP, SAA, calprotectin, age, BMI, obesity, systolic BP, and the use of antihypertensive drugs at baseline were all associated with higher LV mass index at follow up in univariable analyses (all p < 0.05). In multivariable analyses, higher CRP (β 0.21, p = 0.04) at baseline was associated with higher LV mass index at baseline, independent of sex, age and presence of obesity (Table 3). Obesity (β = 0.39, p < 0.001) at baseline was significantly associated with higher LV mass index at follow-up in multivariable analyses, after adjustment for age, sex, CRP and b/tsDMARD therapy at follow-up. Use of bDMARDs (β −0.22, p = 0.03) at follow-up remained associated with lower LV mass index at follow-up after adjustment for age, sex, obesity, and CRP levels in multivariable analyses (Table 3).

**Table 3:** Factors associated with LV mass index in multivariable linear regression analyses at baseline and follow-up.

	LV mass index at baseline R^2^ 0.35, p < 0.001		LV mass index at follow-up R^2^ 0.37, p < 0.001	
	**β**	**P**	**β**	**P**
Age at baseline, years	0.15	0.11	0.18	0.06
Female sex	−0.26	0.008	−0.20	0.04
Obesity at baseline	0.43	<0.001	0.39	<0.001
lnCRP at baseline, μg/ml	0.21	0.04	0.17	0.09
Use of bDMARD at follow-up	–	–	−0.22	0.03

CRP: C-reactive protein; bDMARDs: biological disease modifying antirheumatic drugs; LV: left ventricular; β: standardized beta-coefficient.

## 4 Discussion

In the present study in patients with RA on DMARD therapy, the mean LV mass index did not change during almost two years of follow-up. Obesity and higher levels CRP were associated with higher LV mass index, while the continuous use of bDMARDs during the study period was associated with lower LV mass index.

We did not observe change in LV mass index or the prevalence of LV hypertrophy during treatment with b/tsDMARDs. Previous studies using CMR have shown inconsistent results of bDMARD treatment on LV mass in patients with RA [12,13]. In a British randomized control trial of 81 patients with RA receiving treatment with the tumour necrosis factor alpha inhibitor (TNFi) etanercept and methotrexate versus control subjects, LV mass increased after 2 years of treatment [13]. However, a Japanese study in 20 women with RA found that LV mass decreased after 52 weeks of treatment with the interleukin 6 receptor inhibitor tocilizumab compared to controls [12]. Inconsistent results regarding the effect of bDMARD treatment on LV mass index have also been seen in echocardiographic studies in patients with RA [14,28]. In a study of 48 patients with RA, treatment with etanercept over 6 months was associated with significant reduction in LV mass index compared to treatment with methotrexate alone [14]. However, in another study in 33 patients with RA, no difference in LV mass index was found after 6 months of TNFi treatment [28]. Differences in follow-up length and patient's characteristics such as age and CVD risk factors likely contributed to the differences between the studies. Few studies have conducted longitudinal assessment of LV structure in patients with RA using echocardiography. In an American study that included 160 patients with RA and 1,391 control subjects, a decrease in LV mass was found in both groups over 5 years follow-up [29]. However, only 19% of patients in that study used bDMARD therapy. Further, the numerical difference in LV mass index was only 3.4 g/m^2^ between baseline and follow-up, and of similar magnitude as in the control subjects. Such a small change in LV mass index is unlikely to reflect clinical significant change [30].

The adverse effect of increased LV mass is well known [31,32]. In animal models, increased LV mass is associated with greater energy spending [33]. In a recent study in almost 1,000 hypertensive patients followed for 10-years, greater increase in LV mass index was associated with cardiovascular mortality, even in individuals who did not meet the criteria for LV hypertrophy [34]. Although the average LV mass index was unchanged during follow-up in the present study, patients that were using bDMARD therapy both at baseline and at follow-up had lower LV mass index at follow-up, supporting the hypothesis that there is potential cardiac benefit of bDMARD treatment that should be tested further. Discontinuation or change in b/tsDMARD treatment is common in clinical practice for a variety of reasons including adverse events and lack of effectiveness [35]. However, because of a limited sample size, we were not able to explore the association between individual types of b/tsDMARD treatment with LV mass index in the current cohort.

In the present study, higher levels of CRP were associated with higher LV mass index. The association between inflammatory markers and higher LV mass index has been little studied in RA patients. Still, our findings are in line with observations from other patient populations [3,[36], [37], [38], [39]]. In patients with psoriasis, an autoimmune, inflammatory skin disorder, we recently demonstrated that CRP and SAA, as well as markers of T cells mediated inflammation were associated with larger LV mass index [36]. CRP was also found to be associated with LV hypertrophy in a cross-sectional study consisting of 705 patients with resistant hypertension [38]. LV hypertrophy identified by electrocardiogram was associated with higher levels of CRP at follow-up 6 years later in a community-based study of 1564 participants [39]. In a study using CMR in 39 patients with RA, increased disease activity correlated with a higher incidence of non-ischemic myocardial fibrosis [3]. Taken together, our findings add to these previous cross-sectional studies that suggest an association between inflammatory markers and LV remodelling.

Obesity was associated with higher LV mass in the present study. This is in line with research in different populations [40,41], including children with obesity [42] and patients with RA [10,43]. In RA, obesity could amplify systemic inflammation, contributing to myocardial stress and fibrosis and promoting LV remodelling. Previously studies have also shown that patients with RA that are obese have higher disease activity and lower remission rates than patients with normal weight, possible due to inadequate treatment doses or increased drug-clearance [44,45]. However, CRP is included in DAS28 which could partly explain the observed higher disease activity in obese patients. In our study, patients with obesity had higher levels of CRP at follow-up, which could indicate inadequate response to treatment, or be associated with the obesity as a low-grade inflammatory state. These finding underline the health benefits of maintaining normal body weight for patients with RA [46,47].

### 4.1 Study strength and limitation

There are some limitations to this study. Due to the observational design, the study cannot identify causal relationships, and residual confounding might be present. The study did not include a control group and could therefore not document whether LV mass index differs between patients with RA and control persons. Because of the Covid 19 pandemic, sample size was reduced in the study follow-up, since many patients avoided travelling and hospital visits. This has reduced the statistical power in the study. Information on cumulative exposure to DMARDs prior to study inclusion is not available in the study. Furthermore, this cohort consists of RA patients with insufficient response to previous DMARD therapy from a limited geographical area, generalizability of the findings to more diverse populations should be done with caution. The use of a core laboratory for echocardiographic analyses, as recommended by guidelines represents a strength of the present study [23].

## 5 Conclusion

In this prospective observational study in patients with RA on DMARD treatment, the mean LV mass index remained stable during 22 months of follow-up. Obesity emerged as the major factor associated with higher LV mass index, while use of bDMARD throughout the study period was associated with lower LV mass index. These findings support a multifactorial approach for promoting cardiac health in patients with RA.

## CRediT authorship contribution statement

**Anja Linde:** Writing – review & editing, Writing – original draft, Formal analysis, Data curation, Conceptualization. **Eva Gerdts:** Writing – review & editing, Funding acquisition, Data curation, Conceptualization. **Bjørg T. Fevang:** Writing – review & editing, Data curation. **Arve Ulvik:** Writing – review & editing, Data curation. **Per M. Ueland:** Writing – review & editing, Data curation. **Klaus Meyer:** Writing – review & editing, Data curation. **Ester Kringeland:** Writing – review & editing, Data curation. **Helga Midtbø:** Writing – review & editing, Writing – original draft, Funding acquisition, Formal analysis, Data curation, Conceptualization.

## Disclosure statement

The authors declare no conflict of interest.

## Funding

This work was supported by Revmatikernes Forskningsfond Marit Hanssens Minnefond and the Western and South-Eastern Regional Health Authorities of Norway.

## Declaration of competing interest

The authors report no conflicts of interests.

## Data Availability

The participants in this study did not consent to share data publicly, supporting data is therefore not available.
